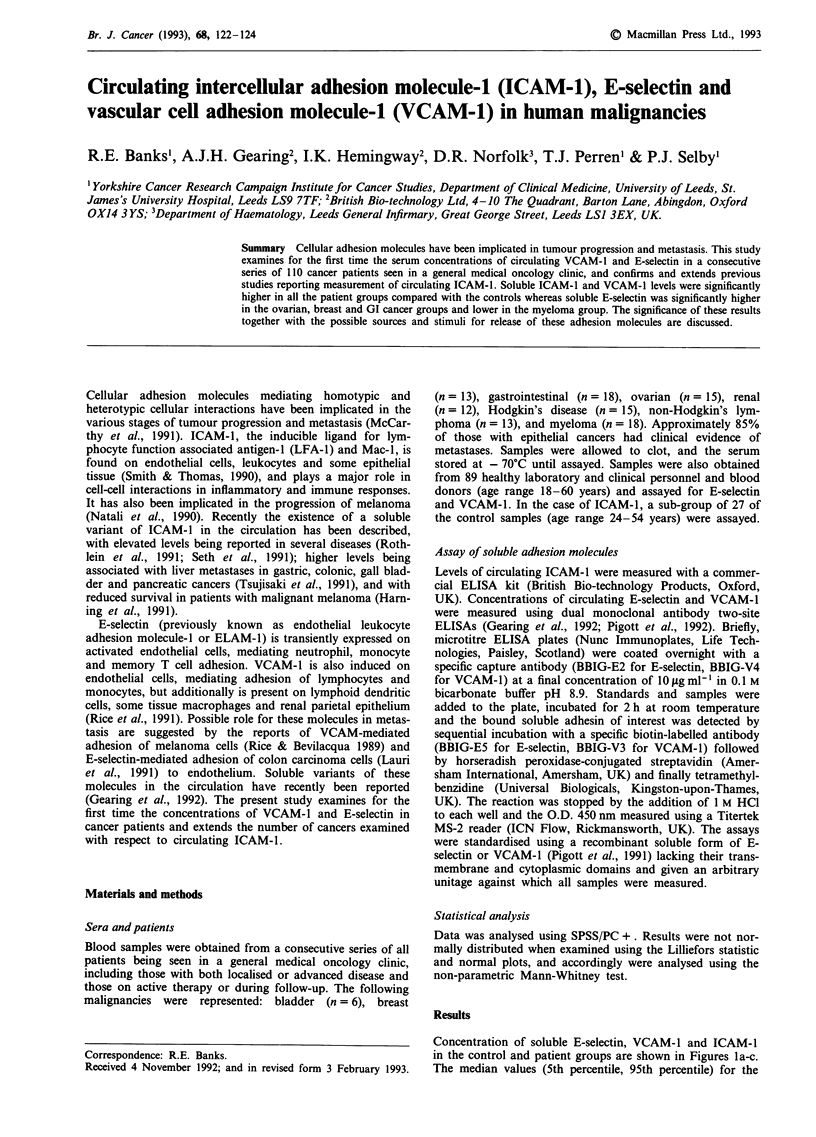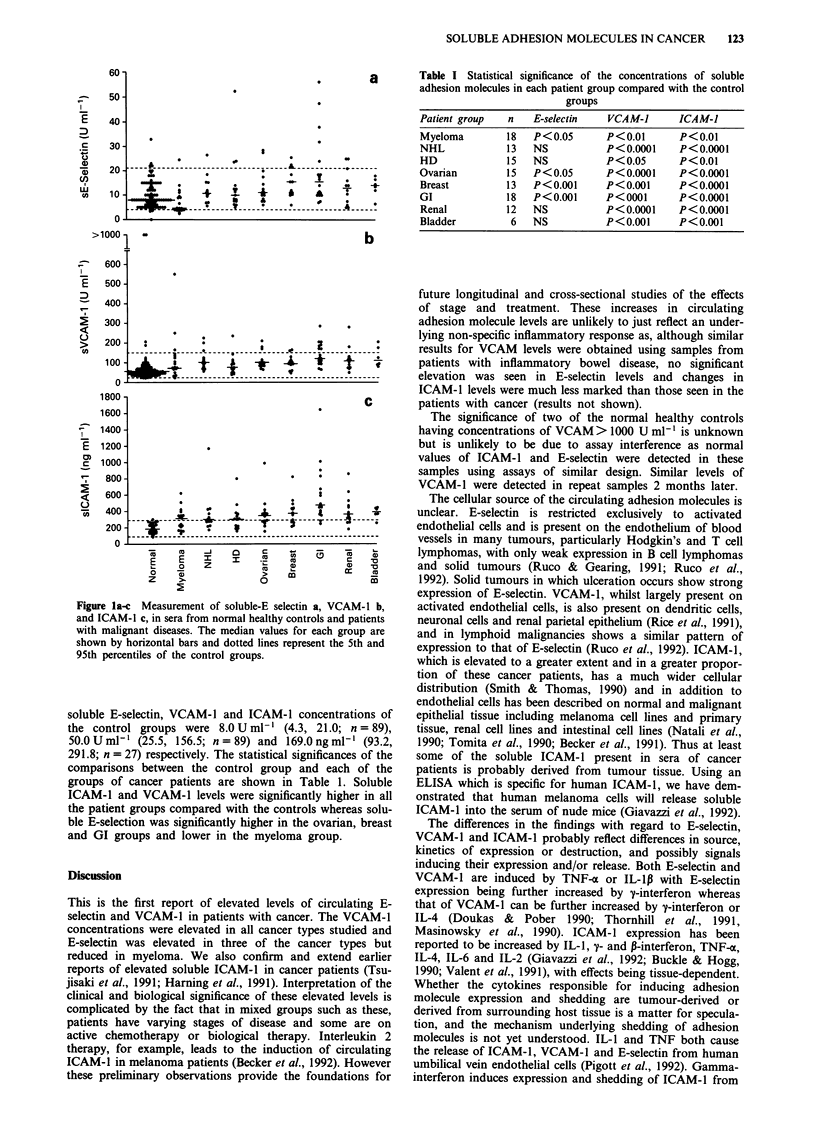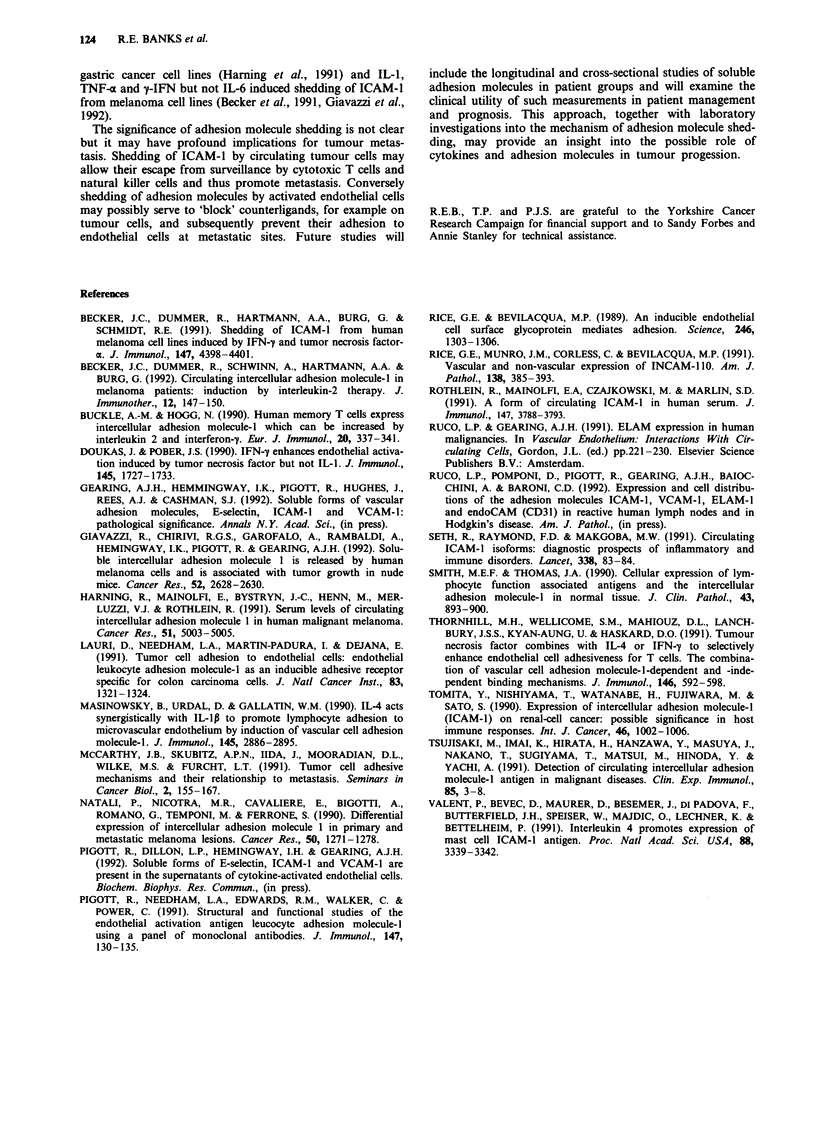# Circulating intercellular adhesion molecule-1 (ICAM-1), E-selectin and vascular cell adhesion molecule-1 (VCAM-1) in human malignancies.

**DOI:** 10.1038/bjc.1993.298

**Published:** 1993-07

**Authors:** R. E. Banks, A. J. Gearing, I. K. Hemingway, D. R. Norfolk, T. J. Perren, P. J. Selby

**Affiliations:** Yorkshire Cancer Research Campaign Institute for Cancer Studies, Department of Clinical Medicine, University of Leeds, St. James's University Hospital, UK.

## Abstract

Cellular adhesion molecules have been implicated in tumour progression and metastasis. This study examines for the first time the serum concentrations of circulating VCAM-1 and E-selectin in a consecutive series of 110 cancer patients seen in a general medical oncology clinic, and confirms and extends previous studies reporting measurement of circulating ICAM-1. Soluble ICAM-1 and VCAM-1 levels were significantly higher in all the patient groups compared with the controls whereas soluble E-selectin was significantly higher in the ovarian, breast and GI cancer groups and lower in the myeloma group. The significance of these results together with the possible sources and stimuli for release of these adhesion molecules are discussed.


 © Macmillan Press Ltd., 1993

### Circulating intercellular adhesion molecule-1 (ICAM-1), E-selectin and vascular cell adhesion molecule-1 (VCAM-1) in human malignancies

R.E. Banks[1], A.J.H. Gearing[2], I.K. Hemingway[2], D.R. Norfolk[3], T.J. Perren[1] & P.J. Selby[1]

[1]*Yorkshire Cancer Research Campaign Institute for Cancer Studies, Department of Clinical Medicine, University of Leeds, St. James's University Hospital, Leeds LS9 7TF;* [2]*British Bio-technology Ltd, 4-10 The Quadrant, Barton Lane, Abingdon, Oxford OX14 3YS;* [3]*Department of Haematology, Leeds General Infirmary, Great George Street, Leeds LS1 3EX, UK.*

**Summary** Cellular adhesion molecules have been implicated in tumour progression and metastasis. This study examines for the first time the serum concentrations of circulating VCAM-1 and E-selectin in a consecutive series of 110 cancer patients seen in a general medical oncology clinic, and confirms and extends previous studies reporting measurement of circulating ICAM-1. Soluble ICAM-1 and VCAM-1 levels were significantly higher in all the patient groups compared with the controls whereas soluble E-selectin was significantly higher in the ovarian, breast and GI cancer groups and lower in the myeloma group. The significance of these results together with the possible sources and stimuli for release of these adhesion molecules are discussed.

Cellular adhesion molecules mediating homotypic and heterotypic cellular interactions have been implicated in the various stages of tumour progression and metastasis (McCarthy *et al.*, 1991). ICAM-1, the inducible ligand for lymphocyte function associated antigen-1 (LFA-1) and Mac-1, is found on endothelial cells, leukocytes and some epithelial tissue (Smith & Thomas, 1990), and plays a major role in cell-cell interactions in inflammatory and immune responses. It has also been implicated in the progression of melanoma (Natali *et al.*, 1990). Recently the existence of a soluble variant of ICAM-1 in the circulation has been described, with elevated levels being reported in several diseases (Rothlein *et al.*, 1991; Seth *et al.*, 1991); higher levels being associated with liver metastases in gastric, colonic, gall bladder and pancreatic cancers (Tsujisaki *et al.*, 1991), and with reduced survival in patients with malignant melanoma (Harning *et al.*, 1991).

E-selectin (previously known as endothelial leukocyte adhesion molecule-1 or ELAM-1) is transiently expressed on activated endothelial cells, mediating neutrophil, monocyte and memory T cell adhesion. VCAM-1 is also induced on endothelial cells, mediating adhesion of lymphocytes and monocytes, but additionally is present on lymphoid dendritic cells, some tissue macrophages and renal parietal epithelium (Rice *et al.*, 1991). Possible role for these molecules in metastasis are suggested by the reports of VCAM-mediated adhesion of melanoma cells (Rice & Bevilacqua 1989) and E-selectin-mediated adhesion of colon carcinoma cells (Lauri *et al.*, 1991) to endothelium. Soluble variants of these molecules in the circulation have recently been reported (Gearing *et al.*, 1992). The present study examines for the first time the concentrations of VCAM-1 and E-selectin in cancer patients and extends the number of cancers examined with respect to circulating ICAM-1.

### Materials and methods

### *Sera and patients*

Blood samples were obtained from a consecutive series of all patients being seen in a general medical oncology clinic, including those with both localised or advanced disease and those on active therapy or during follow-up. The following malignancies were represented: bladder ($n = 6$), breast ($n = 13$), gastrointestinal ($n = 18$), ovarian ($n = 15$), renal ($n = 12$), Hodgkin's disease ($n = 15$), non-Hodgkin's lymphoma ($n = 13$), and myeloma ($n = 18$). Approximately 85% of those with epithelial cancers had clinical evidence of metastases. Samples were allowed to clot, and the serum stored at $-70°C$ until assayed. Samples were also obtained from 89 healthy laboratory and clinical personnel and blood donors (age range 18-60 years) and assayed for E-selectin and VCAM-1. In the case of ICAM-1, a sub-group of 27 of the control samples (age range 24-54 years) were assayed.

### *Assay of soluble adhesion molecules*

Levels of circulating ICAM-1 were measured with a commercial ELISA kit (British Bio-technology Products, Oxford, UK). Concentrations of circulating E-selectin and VCAM-1 were measured using dual monoclonal antibody two-site ELISAs (Gearing *et al.*, 1992; Pigott *et al.*, 1992). Briefly, microtitre ELISA plates (Nunc Immunoplates, Life Technologies, Paisley, Scotland) were coated overnight with a specific capture antibody (BBIG-E2 for E-selectin, BBIG-V4 for VCAM-1) at a final concentration of $10\,\mu g\,ml^{-1}$ in 0.1 M bicarbonate buffer pH 8.9. Standards and samples were added to the plate, incubated for 2 h at room temperature and the bound soluble adhesin of interest was detected by sequential incubation with a specific biotin-labelled antibody (BBIG-E5 for E-selectin, BBIG-V3 for VCAM-1) followed by horseradish peroxidase-conjugated streptavidin (Amersham International, Amersham, UK) and finally tetramethylbenzidine (Universal Biologicals, Kingston-upon-Thames, UK). The reaction was stopped by the addition of 1 M HCl to each well and the O.D. 450 nm measured using a Titertek MS-2 reader (ICN Flow, Rickmansworth, UK). The assays were standardised using a recombinant soluble form of E-selectin or VCAM-1 (Pigott *et al.*, 1991) lacking their transmembrane and cytoplasmic domains and given an arbitrary unitage against which all samples were measured.

### *Statistical analysis*

Data was analysed using SPSS/PC+. Results were not normally distributed when examined using the Lilliefors statistic and normal plots, and accordingly were analysed using the non-parametric Mann-Whitney test.

### Results

Concentration of soluble E-selectin, VCAM-1 and ICAM-1 in the control and patient groups are shown in Figures 1a-c. The median values (5th percentile, 95th percentile) for the

Correspondence: R.E. Banks.
Received 4 November 1992; and in revised form 3 February 1993.

**Figure 1a-c** Measurement of soluble-E selectin **a**, VCAM-1 **b**, and ICAM-1 **c**, in sera from normal healthy controls and patients with malignant diseases. The median values for each group are shown by horizontal bars and dotted lines represent the 5th and 95th percentiles of the control groups.

soluble E-selectin, VCAM-1 and ICAM-1 concentrations of the control groups were 8.0 U ml$^{-1}$ (4.3, 21.0; $n = 89$), 50.0 U ml$^{-1}$ (25.5, 156.5; $n = 89$) and 169.0 ng ml$^{-1}$ (93.2, 291.8; $n = 27$) respectively. The statistical significances of the comparisons between the control group and each of the groups of cancer patients are shown in Table 1. Soluble ICAM-1 and VCAM-1 levels were significantly higher in all the patient groups compared with the controls whereas soluble E-selection was significantly higher in the ovarian, breast and GI groups and lower in the myeloma group.

### Discussion

This is the first report of elevated levels of circulating E-selectin and VCAM-1 in patients with cancer. The VCAM-1 concentrations were elevated in all cancer types studied and E-selectin was elevated in three of the cancer types but reduced in myeloma. We also confirm and extend earlier reports of elevated soluble ICAM-1 in cancer patients (Tsujisaki *et al.*, 1991; Harning *et al.*, 1991). Interpretation of the clinical and biological significance of these elevated levels is complicated by the fact that in mixed groups such as these, patients have varying stages of disease and some are on active chemotherapy or biological therapy. Interleukin 2 therapy, for example, leads to the induction of circulating ICAM-1 in melanoma patients (Becker *et al.*, 1992). However these preliminary observations provide the foundations for

**Table I** Statistical significance of the concentrations of soluble adhesion molecules in each patient group compared with the control groups

| *Patient group* | *n* | *E-selectin* | *VCAM-1* | *ICAM-1* |
|---|---|---|---|---|
| Myeloma | 18 | $P<0.05$ | $P<0.01$ | $P<0.01$ |
| NHL | 13 | NS | $P<0.0001$ | $P<0.0001$ |
| HD | 15 | NS | $P<0.05$ | $P<0.01$ |
| Ovarian | 15 | $P<0.05$ | $P<0.0001$ | $P<0.0001$ |
| Breast | 13 | $P<0.001$ | $P<0.001$ | $P<0.0001$ |
| GI | 18 | $P<0.001$ | $P<0001$ | $P<0.0001$ |
| Renal | 12 | NS | $P<0.0001$ | $P<0.0001$ |
| Bladder | 6 | NS | $P<0.001$ | $P<0.001$ |

future longitudinal and cross-sectional studies of the effects of stage and treatment. These increases in circulating adhesion molecule levels are unlikely to just reflect an underlying non-specific inflammatory response as, although similar results for VCAM levels were obtained using samples from patients with inflammatory bowel disease, no significant elevation was seen in E-selectin levels and changes in ICAM-1 levels were much less marked than those seen in the patients with cancer (results not shown).

The significance of two of the normal healthy controls having concentrations of VCAM > 1000 U ml$^{-1}$ is unknown but is unlikely to be due to assay interference as normal values of ICAM-1 and E-selectin were detected in these samples using assays of similar design. Similar levels of VCAM-1 were detected in repeat samples 2 months later.

The cellular source of the circulating adhesion molecules is unclear. E-selectin is restricted exclusively to activated endothelial cells and is present on the endothelium of blood vessels in many tumours, particularly Hodgkin's and T cell lymphomas, with only weak expression in B cell lymphomas and solid tumours (Ruco & Gearing, 1991; Ruco *et al.*, 1992). Solid tumours in which ulceration occurs show strong expression of E-selectin. VCAM-1, whilst largely present on activated endothelial cells, is also present on dendritic cells, neuronal cells and renal parietal epithelium (Rice *et al.*, 1991), and in lymphoid malignancies shows a similar pattern of expression to that of E-selectin (Ruco *et al.*, 1992). ICAM-1, which is elevated to a greater extent and in a greater proportion of these cancer patients, has a much wider cellular distribution (Smith & Thomas, 1990) and in addition to endothelial cells has been described on normal and malignant epithelial tissue including melanoma cell lines and primary tissue, renal cell lines and intestinal cell lines (Natali *et al.*, 1990; Tomita *et al.*, 1990; Becker *et al.*, 1991). Thus at least some of the soluble ICAM-1 present in sera of cancer patients is probably derived from tumour tissue. Using an ELISA which is specific for human ICAM-1, we have demonstrated that human melanoma cells will release soluble ICAM-1 into the serum of nude mice (Giavazzi *et al.*, 1992).

The differences in the findings with regard to E-selectin, VCAM-1 and ICAM-1 probably reflect differences in source, kinetics of expression or destruction, and possibly signals inducing their expression and/or release. Both E-selectin and VCAM-1 are induced by TNF-α or IL-1β with E-selectin expression being further increased by γ-interferon whereas that of VCAM-1 can be further increased by γ-interferon or IL-4 (Doukas & Pober 1990; Thornhill *et al.*, 1991, Masinowsky *et al.*, 1990). ICAM-1 expression has been reported to be increased by IL-1, γ- and β-interferon, TNF-α, IL-4, IL-6 and IL-2 (Giavazzi *et al.*, 1992; Buckle & Hogg, 1990; Valent *et al.*, 1991), with effects being tissue-dependent. Whether the cytokines responsible for inducing adhesion molecule expression and shedding are tumour-derived or derived from surrounding host tissue is a matter for speculation, and the mechanism underlying shedding of adhesion molecules is not yet understood. IL-1 and TNF both cause the release of ICAM-1, VCAM-1 and E-selectin from human umbilical vein endothelial cells (Pigott *et al.*, 1992). Gamma-interferon induces expression and shedding of ICAM-1 from gastric cancer cell lines (Harning et al., 1991) and IL-1, TNF-α and γ-IFN but not IL-6 induced shedding of ICAM-1 from melanoma cell lines (Becker et al., 1991, Giavazzi et al., 1992).

The significance of adhesion molecule shedding is not clear but it may have profound implications for tumour metastasis. Shedding of ICAM-1 by circulating tumour cells may allow their escape from surveillance by cytotoxic T cells and natural killer cells and thus promote metastasis. Conversely shedding of adhesion molecules by activated endothelial cells may possibly serve to 'block' counterligands, for example on tumour cells, and subsequently prevent their adhesion to endothelial cells at metastatic sites. Future studies will include the longitudinal and cross-sectional studies of soluble adhesion molecules in patient groups and will examine the clinical utility of such measurements in patient management and prognosis. This approach, together with laboratory investigations into the mechanism of adhesion molecule shedding, may provide an insight into the possible role of cytokines and adhesion molecules in tumour progession.

R.E.B., T.P. and P.J.S. are grateful to the Yorkshire Cancer Research Campaign for financial support and to Sandy Forbes and Annie Stanley for technical assistance.

### References

BECKER, J.C., DUMMER, R., HARTMANN, A.A., BURG, G. & SCHMIDT, R.E. (1991). Shedding of ICAM-1 from human melanoma cell lines induced by IFN-γ and tumor necrosis factor-α. *J. Immunol.*, **147**, 4398-4401.

BECKER, J.C., DUMMER, R., SCHWINN, A., HARTMANN, A.A. & BURG, G. (1992). Circulating intercellular adhesion molecule-1 in melanoma patients: induction by interleukin-2 therapy. *J. Immunother.*, **12**, 147-150.

BUCKLE, A.-M. & HOGG, N. (1990). Human memory T cells express intercellular adhesion molecule-1 which can be increased by interleukin 2 and interferon-γ. *Eur. J. Immunol.*, **20**, 337-341.

DOUKAS, J. & POBER, J.S. (1990). IFN-γ enhances endothelial activation induced by tumor necrosis factor but not IL-1. *J. Immunol.*, **145**, 1727-1733.

GEARING, A.J.H., HEMMINGWAY, I.K., PIGOTT, R., HUGHES, J., REES, A.J. & CASHMAN, S.J. (1992). Soluble forms of vascular adhesion molecules, E-selectin, ICAM-1 and VCAM-1: pathological significance. *Annals N.Y. Acad. Sci.*, (in press).

GIAVAZZI, R., CHIRIVI, R.G.S., GAROFALO, A., RAMBALDI, A., HEMINGWAY, I.K., PIGOTT, R. & GEARING, A.J.H. (1992). Soluble intercellular adhesion molecule 1 is released by human melanoma cells and is associated with tumor growth in nude mice. *Cancer Res.*, **52**, 2628-2630.

HARNING, R., MAINOLFI, E., BYSTRYN, J.-C., HENN, M., MERLUZZI, V.J. & ROTHLEIN, R. (1991). Serum levels of circulating intercellular adhesion molecule 1 in human malignant melanoma. *Cancer Res.*, **51**, 5003-5005.

LAURI, D., NEEDHAM, L.A., MARTIN-PADURA, I. & DEJANA, E. (1991). Tumor cell adhesion to endothelial cells: endothelial leukocyte adhesion molecule-1 as an inducible adhesive receptor specific for colon carcinoma cells. *J. Natl Cancer Inst.*, **83**, 1321-1324.

MASINOWSKY, B., URDAL, D. & GALLATIN, W.M. (1990). IL-4 acts synergistically with IL-1β to promote lymphocyte adhesion to microvascular endothelium by induction of vascular cell adhesion molecule-1. *J. Immunol.*, **145**, 2886-2895.

MCCARTHY, J.B., SKUBITZ, A.P.N., IIDA, J., MOORADIAN, D.L., WILKE, M.S. & FURCHT, L.T. (1991). Tumor cell adhesive mechanisms and their relationship to metastasis. *Seminars in Cancer Biol.*, **2**, 155-167.

NATALI, P., NICOTRA, M.R., CAVALIERE, E., BIGOTTI, A., ROMANO, G., TEMPONI, M. & FERRONE, S. (1990). Differential expression of intercellular adhesion molecule 1 in primary and metastatic melanoma lesions. *Cancer Res.*, **50**, 1271-1278.

PIGOTT, R., DILLON, L.P., HEMINGWAY, I.H. & GEARING, A.J.H. (1992). Soluble forms of E-selectin, ICAM-1 and VCAM-1 are present in the supernatants of cytokine-activated endothelial cells. *Biochem. Biophys. Res. Commun.*, (in press).

PIGOTT, R., NEEDHAM, L.A., EDWARDS, R.M., WALKER, C. & POWER, C. (1991). Structural and functional studies of the endothelial activation antigen leucocyte adhesion molecule-1 using a panel of monoclonal antibodies. *J. Immunol.*, **147**, 130-135.

RICE, G.E. & BEVILACQUA, M.P. (1989). An inducible endothelial cell surface glycoprotein mediates adhesion. *Science*, **246**, 1303-1306.

RICE, G.E., MUNRO, J.M., CORLESS, C. & BEVILACQUA, M.P. (1991). Vascular and non-vascular expression of INCAM-110. *Am. J. Pathol.*, **138**, 385-393.

ROTHLEIN, R., MAINOLFI, E.A, CZAJKOWSKI, M. & MARLIN, S.D. (1991). A form of circulating ICAM-1 in human serum. *J. Immunol.*, 147, 3788-3793.

RUCO, L.P. & GEARING, A.J.H. (1991). ELAM expression in human malignancies. In *Vascular Endothelium: Interactions With Circulating Cells*, Gordon, J.L. (ed.) pp.221-230. Elsevier Science Publishers B.V.: Amsterdam.

RUCO, L.P., POMPONI, D., PIGOTT, R., GEARING, A.J.H., BAIOCCHINI, A. & BARONI, C.D. (1992). Expression and cell distributions of the adhesion molecules ICAM-1, VCAM-1, ELAM-1 and endoCAM (CD31) in reactive human lymph nodes and in Hodgkin's disease. *Am. J. Pathol.*, (in press).

SETH, R., RAYMOND, F.D. & MAKGOBA, M.W. (1991). Circulating ICAM-1 isoforms: diagnostic prospects of inflammatory and immune disorders. *Lancet*, **338**, 83-84.

SMITH, M.E.F. & THOMAS, J.A. (1990). Cellular expression of lymphocyte function associated antigens and the intercellular adhesion molecule-1 in normal tissue. *J. Clin. Pathol.*, **43**, 893-900.

THORNHILL, M.H., WELLICOME, S.M., MAHIOUZ, D.L., LANCHBURY, J.S.S., KYAN-AUNG, U. & HASKARD, D.O. (1991). Tumour necrosis factor combines with IL-4 or IFN-γ to selectively enhance endothelial cell adhesiveness for T cells. The combination of vascular cell adhesion molecule-1-dependent and -independent binding mechanisms. *J. Immunol.*, **146**, 592-598.

TOMITA, Y., NISHIYAMA, T., WATANABE, H., FUJIWARA, M. & SATO, S. (1990). Expression of intercellular adhesion molecule-1 (ICAM-1) on renal-cell cancer: possible significance in host immune responses. *Int. J. Cancer*, **46**, 1002-1006.

TSUJISAKI, M., IMAI, K., HIRATA, H., HANZAWA, Y., MASUYA, J., NAKANO, T., SUGIYAMA, T., MATSUI, M., HINODA, Y. & YACHI, A. (1991). Detection of circulating intercellular adhesion molecule-1 antigen in malignant diseases. *Clin. Exp. Immunol.*, **85**, 3-8.

VALENT, P., BEVEC, D., MAURER, D., BESEMER, J., DI PADOVA, F., BUTTERFIELD, J.H., SPEISER, W., MAJDIC, O., LECHNER, K. & BETTELHEIM, P. (1991). Interleukin 4 promotes expression of mast cell ICAM-1 antigen. *Proc. Natl Acad. Sci. USA*, **88**, 3339-3342.